# Genome-wide comparative analysis of the *BAHD* superfamily in seven Rosaceae species and expression analysis in pear (*Pyrus bretschneideri*)

**DOI:** 10.1186/s12870-019-2230-z

**Published:** 2020-01-08

**Authors:** Chunxin Liu, Xin Qiao, Qionghou Li, Weiwei Zeng, Shuwei Wei, Xin Wang, Yangyang Chen, Xiao Wu, Jun Wu, Hao Yin, Shaoling Zhang

**Affiliations:** 1 Center of Pear Engineering Technology Research, State Key Laboratory of Crop Genetics and Germplasm Enhancement, Nanjing Agricultural University, Nanjing, 210095 Jiangsu China; 2 Shandong Institute of Pomology, Taian, 271000 Shandong China

**Keywords:** BAHD, Pear, Evolution, Rosaceae, Transcriptome, Volatile esters

## Abstract

**Background:**

The *BAHD* acyltransferase superfamily exhibits various biological roles in plants, including regulating fruit quality, catalytic synthesizing of terpene, phenolics and esters, and improving stress resistance. However, the copy numbers, expression characteristics and associations with fruit aroma formation of the *BAHD* genes remain unclear.

**Results:**

In total, 717 *BAHD* genes were obtained from the genomes of seven Rosaceae, (*Pyrus bretschneideri*, *Malus domestica*, *Prunus avium*, *Prunus persica*, *Fragaria vesca*, *Pyrus communis* and *Rubus occidentalis*). Based on the detailed phylogenetic analysis and classifications in model plants, we divided the *BAHD* family genes into seven groups, I-a, I-b, II-a, II-b, III-a, IV and V. An inter-species synteny analysis revealed the ancient origin of *BAHD* superfamily with 78 syntenic gene pairs were detected among the seven Rosaceae species. Different types of gene duplication events jointly drive the expansion of *BAHD* superfamily, and purifying selection dominates the evolution of *BAHD* genes supported by the small Ka/Ks ratios. Based on the correlation analysis between the ester content and expression levels of *BAHD* genes at different developmental stages, four candidate genes were selected for verification as assessed by qRT-PCR. The result implied that *Pbr020016.1*, *Pbr019034.1*, *Pbr014028.1* and *Pbr029551.1* are important candidate genes involved in aroma formation during pear fruit development.

**Conclusion:**

We have thoroughly identified the *BAHD* superfamily genes and performed a comprehensive comparative analysis of their phylogenetic relationships, expansion patterns, and expression characteristics in seven Rosaceae species, and we also obtained four candidate genes involved in aroma synthesis in pear fruit. These results provide a theoretical basis for future studies of the specific biological functions of *BAHD* superfamily members and the improvement of pear fruit quality.

## Background

Pear is an important temperate Rosaceae fruit tree worldwide. According to historical records, pears originated between 55 and 65 million years ago, and their cultivation history can be traced back for more than 30,000 years [1]. In 2012, the genome sequence of pear (*Pyrus bretschneideri* cv. ‘Dangshansuli’) was released, providing a resource for research on genomics and molecular biology in pear. At present, genome sequences of six other Rosaceae fruit species are also available, allowing comparative genomics studies among economically important Rosaceae species. Fruit quality including sugar content, fruit size, fruit aroma, is tightly coupled with consumer choice and commercial value. Fruit aroma is an inherent quality/characteristic, which distinguishes the different fruit species even different cultivars within a species. In apple, the main aromatic components were 1-hexanol [2]. In red-fleshed peach fruit, fruity note latone γ-hexalactone is the major aroma components [3].In banana, 2-pentyl acetate, 3-methylbutyl acetate were the most important contributors to the aroma [4]. In European pear cultivars, the volatile organic compounds mainly belong to primary esters, alcohols and alkanes [5]. Moreover, it has been pointed out that esters and aldehydes were key volatile compounds shared by 33 cultivars of the Chinese pear *Pyrus ussuriensis* [6]. It can be conclude that the fruit aroma is a complex mixture of a large number of volatile compounds, but the esters seem to be the most extensive.

Four pathways were reported involved in volatile aroma compounds biosynthesis, including fatty acids pathway, amino acid pathway, terpenoids pathway and carotenoid pathway. Among them, fatty acids are major precursors of aroma volatiles in most fruit species [7], fatty acid-derived straight-chain alcohols, aldehydes and esters are important aroma compounds responsible for fresh fruit flavors and are consisted by three processes: α-oxidation, β-oxidation and the lipoxygenase pathway [8]. β-oxidation of fatty acids is the primary biosynthetic process providing alcohols and acyl coenzyme A (CoAs) for ester formation [7]. Previous study showed that fruit aromas volatiles are were formatted through β-oxidation in pear and apple [9]. Multiple enzymes have been reported involved in β-oxidation. For example, acyl CoAs can be reduced by acyl CoA reductase to aldehyde, which was continually reduced by alcohol dehydrogenase to alcohol, finally, the alcohols were used to produce esters by alcohol acyltransferase (*AAT*) [10]. Moreover, in amino acid pathway, these amino acids can also be the precursors of acyl-CoAs, involved in alcohol esterification reactions catalyzed by *AATs* [11]. Once the basic skeletons were produced through these pathways, the diversity of these volatile compounds can be achieved via acylation, decarboxylation, glycosylation, oxidation/reduction, hydroxylation and methylation, which expand the basic skeletons and modify enzymes [12].

Acylation is an important process of modification of secondary metabolites in plant growth and development. *BAHD* acyltransferase family mainly uses coenzyme A thioester as acyl donor and uses alcohols or amines as receptors to catalyze acylation to form all kinds of acylation products, including lignin monomers, anthocyanins, terpenoids, esters and so on [13–15]. The *BAHD* (benzylalcohol O-acetyl transferase, anthocyanin O-hydroxycinnamoyl transferase, N-hydroxycinnamoyl anthranilate benzoyl transferase and deacetylvindoline 4-O-acetyltransferase [16]) superfamily is composed of enzymes having two common domains (HXXXD and DFGWG) and similar amino acid sequences [17]. The HXXXD motif region is located in the reaction channel center and participates in catalysis. As an indispensable motif for the reaction, the DFGWG motif is located far from the active site [18]. Benzylalcohol O-acetyl transferase identified from the Californian wildflower *Clarkia breweri* can produce the floral volatile benzylacetate [13, 19], deacetylvindoline 4-O-acetyltransferase identified from *Catharanthus roseus* is related to the synthesis of the alkaloid vindoline [13, 20], N-hydroxycinnamoyl anthranilate benzoyl transferase identified from *Dianthus caryophyllus* is responsible for producing a class of phytoalexins known as anthramides [21], and anthocyanin O-hydroxycinnamoyl transferases identified from *Gentiana triflora* can catalyze anthocyanin synthesis [22, 23].

In recent years, members of the *BAHD* acyltransferase family related to ester metabolism have been discovered, such as the *AAT* genes *MpAAT1* in apple [24], *FaAAT2* and *SAAT* in strawberries [25, 26], they play a role in the final step of ester biosynthesis, catalyzing the production of ester compounds with coenzyme A thioester as the donor and alcohol as the receptor. The potential roles of *BAHD* acyltransferase family members in fruit ester synthesis need to be further investigated in pear and other fruit species. Hence, systematically identifying *BAHD* gene family in pear and screening candidate genes that regulate the synthesis of esters are of great significance for artificially regulating the content of pear esters and improving the quality of pears. In this study, we aimed to identify the repertoires of *BAHD* superfamily members in the genomes of pear and six other Rosaceae fruit species. In order to unravel the evolution and expansion mechanisms of *BAHD* superfamily, and screen candidate *BAHD* acyltransferases related to fruit aroma biosynthesis especially ester synthesis, we performed comprehensive analysis on evolutionary and expression patterns using genome and transcriptome resources. Gene structure, conserved motifs, phylogeny, gene duplication, selection pressure and spatiotemporal expression profiles of *BAHD* genes were analyzed in this study. Furthermore, we verified gene expression patterns by qRT-PCR, and several candidate *BAHD* genes that are closely associated with the pear volatile ester content were determined. These results provide insights into the evolution, expansion and functional roles of the *BAHD* superfamily and will aid in further studies of their molecular functions in these fruit species.

## Results

### Identification of *BAHD* genes in seven Rosaceae species

The *BAHD* superfamily’s characteristic domain (Pfam: PF02458) and the *BAHD* Hidden Markov Model (HMM) configuration file (PF02458) were used to identify the *BAHD* members. The online site SMART (http://smart.embl-heidelberg.de/) was used to analyze protein sequences of candidate genes and to determine the presence of the *BAHD* domain. As a result, 773 putative *BAHD* family candidate genes were obtained in the seven species with E-values <1e^− 10^. Furthermore, a multiple sequence alignment was conducted to verify the presence of two characteristic conserved domains (HXXXD and DFGWG) in the *BAHD* family genes [17]. Because they lacked the two domains, three, six, six, six, seven, eight and 20 genes were removed from *Prunus persica* (peach), *P. bretschneideri* (Chinese white pear), *Pyrus communis* (European pear), *Rubus occidentalis* (black raspberry), *Fragaria vesca* (strawberry), *Malus domestica* (apple) and *Prunus avium* (sweet cherry), respectively. Finally, 56 sequences were removed, 717 *BAHD* genes were identified and analyzed (Table 1). Detailed information on the features of *BAHD* genes is available in Additional file 1: Table S1.

**Table 1:** Genomic information and identified *BAHD* gene numbers in Rosaceae species

Common name	Scientific name	Chromosome number (2n)	Release version	Genome gene number	Identified *BAHD* genes
Chinese white pear	*Pyrus bretschneideri*	34	NJAU, v1.0	42,341	114 (120)
Apple	*Malus domestica*	34	JGI, v1.1	63,541	141 (149)
Strawberry	*Fragaria vesca*	14	GDR, v4.0	32,831	89 (96)
European pear	*Pyrus communis*	34	GDR, v2.0	37,445	97 (103)
Sweet cherry	*Prunus avium*	16	GDR, v1.0	43,679	125 (145)
Peach	*Prunus persica*	16	JGI, v1.1	27,864	82 (85)
Black raspberry	*Rubus occidentalis*	14	GDR, v3.0	33,286	69 (75)

The database addresses were listed below: NJAU (http://peargenome.njau.edu.cn/); GDR (http://www.rosaceae.org/); JGI (http://www.jgi.doe.gov/); The numbers in parentheses show the count of genes before filtering for unanchored and missing conserved domain genes

### Phylogenetic and conserved motif analyses of *BAHD* genes in Chinese white pear

**Fig. 1:**
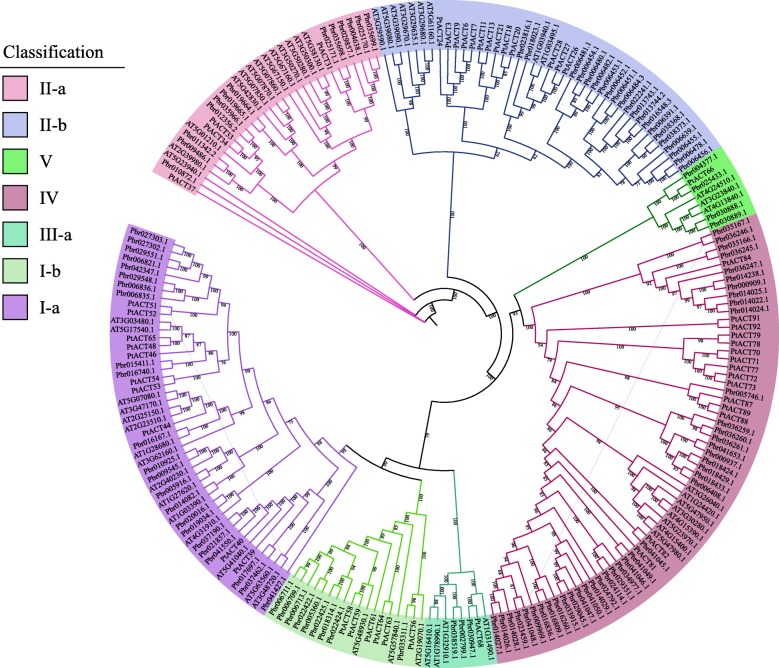
Phylogenetic analysis of *BAHD*s from *Arabidopsis,* Chinese white pear and *Poplar.* The maximum-likelihood method was used to construct the phylogenetic tree

The amino acid sequences encoded by *BAHD* genes in Chinese white pear, *Arabidopsis* and *Populus* were used to construct a phylogenetic tree with maximum-likelihood (ML) method. The Chinese white pear *BAHD* family protein genes were classified into five clades (I, II, III-a, IV and V; Fig. 1). Clade I consisted of two subclades (clades I-a and I-b). The *Arabidopsis* genes belonging to clade I-a are involved in modifying aromatic and aliphatic alcohols in *Arabidopsis* and *Populus* [27, 28]; therefore, we speculated that the *BAHD* genes clustered into clade I-a in Chinese white pear might encode proteins with similar functions. The members of clade I-b had functions related to the biosynthesis of lignin monomeric intermediates [29, 30], including tobacco and *Arabidopsis* shikimate hydroxycinnamoyltransferases [31]. Clade II consisted of two subclades (clades II-a and II-b). The functions of the *Arabidopsis* genes belonging to clade II-a are unknown. Clade II-b contained two *Arabidopsis* genes, *AT3G29590.1* (*At5MAT*) and *AT1G03940.1* (*At3AT1*), which are associated with anthocyanin biosynthesis [15, 32]. Clade III-a contained few members, including four genes from Chinese white pear, three genes from *Arabidopsis*, and one gene from poplar. Clade V contained nine members, including one well-studied *Arabidopsis* gene *AT4G24510.1* (*CER2*) involved in regulating the cuticular wax biosynthesis [33]. Clade IV contained many members involved in catalyzing the acetylation of aromatic alcohols and acetylating small- or medium-chain alcohols [28]. Additionally, only three and four members clustered in clades III-a and V. However, ~ 28.1% (32 of 114) *BAHD* genes were in clade I and 36.0% (41 of 114) were in clade IV, respectively. *BAHD* genes closely related to pear volatile ester contents, such as *AAT*, belonged to clades I and IV.

**Fig. 2:**
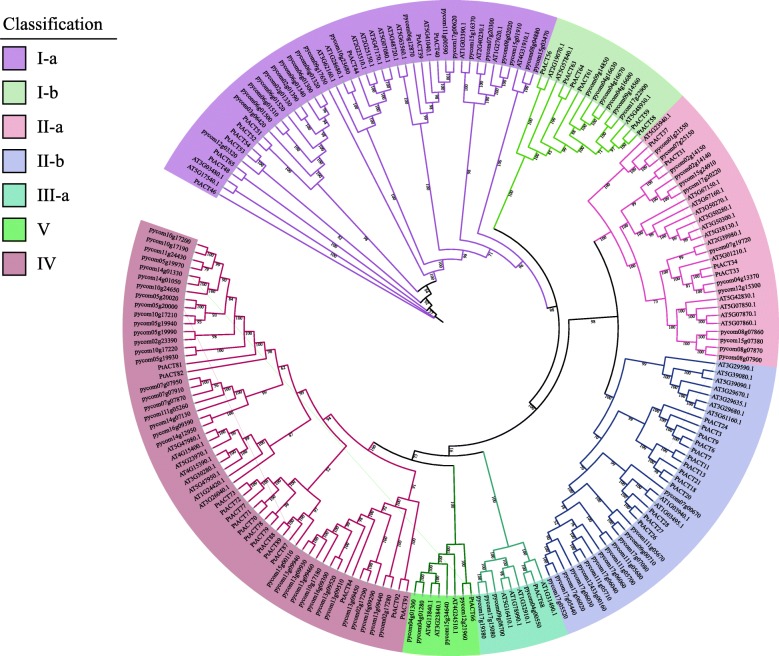
Phylogenetic analysis of *BAHD* from *Arabidopsis, Pyrus communis* and *Populus.* Using the maximum-likelihood method to construct the phylogenetic tree. The amino-acid sequences of *Arabidopsis* and *Populus* were obtained from phytozome (https://phytozome.jgi.doe.gov/pz/portal.html#)

The ML phylogenetic tree of *BAHDs* for European pear with *Arabidopsis* and *Populus* as outgroups was also constructed (Fig. 2). Paralleling the phylogenetic tree built for Chinese white pear, the European pear’s *BAHD* genes were divided into five clades. Clades V and III-a only contained four members, respectively. However, ~ 36.1% (35 of 97) genes clustered in clade IV, and 26.8% (26 of 97) clustered in clade I. Additionally, five function-known acyltransferases of *Arabidopsis* [*AT3G0480.1* (*CHAT*), *AT2G23510.1* (*SDT*), *AT5G48930.1* (*AtHCT*), *AT3G29590.1* (*At5MAT*) and *AT1G03490.1* (*At3AT1*)] were classified into two subgroups (I-a and II-b). These results provided putative candidates for the study of gene functions.

Furthermore, in order to verify the accuracy of the classification of the phylogenetic analysis of the ML method, we also constructed the tree using the neighbor-joining (NJ) method (Additional file 2: Figure S1; Additional file 3: Figure S2). The results show that the NJ phylogenetic tree can also be divided into five clades. In European pear, the type and numbers of the subclades was consistent with the ML phylogenetic tree. In the NJ phylogenetic tree of Chinese white pear, *Pbr034977.1* was clustered in clade II-b, *Pbr029351.1* was clustered in clade V, however, in ML phylogenetic tree of Chinese white pear, these two members were classified in class IV with a bootstrap value of 100, so we speculate that these two members might belong to clade IV. Similarly, three members (*AT4G31910.1*, *Pbr020016.1*, *Pbr019034.1*) in subclade I-b in NJ tree might belong to subclade I-a, since the bootstrap value of the ML tree is higher than that of NJ tree. In addition to the above genes, other genes were divided into the same subclades in both the NJ method and the ML method of Chinese white pear. Moreover, we also found that the bootstrap value of all branches of ML phylogenetic tree was higher than 50, which further indicated the reliability of the classification in ML tree.

**Fig. 3:**
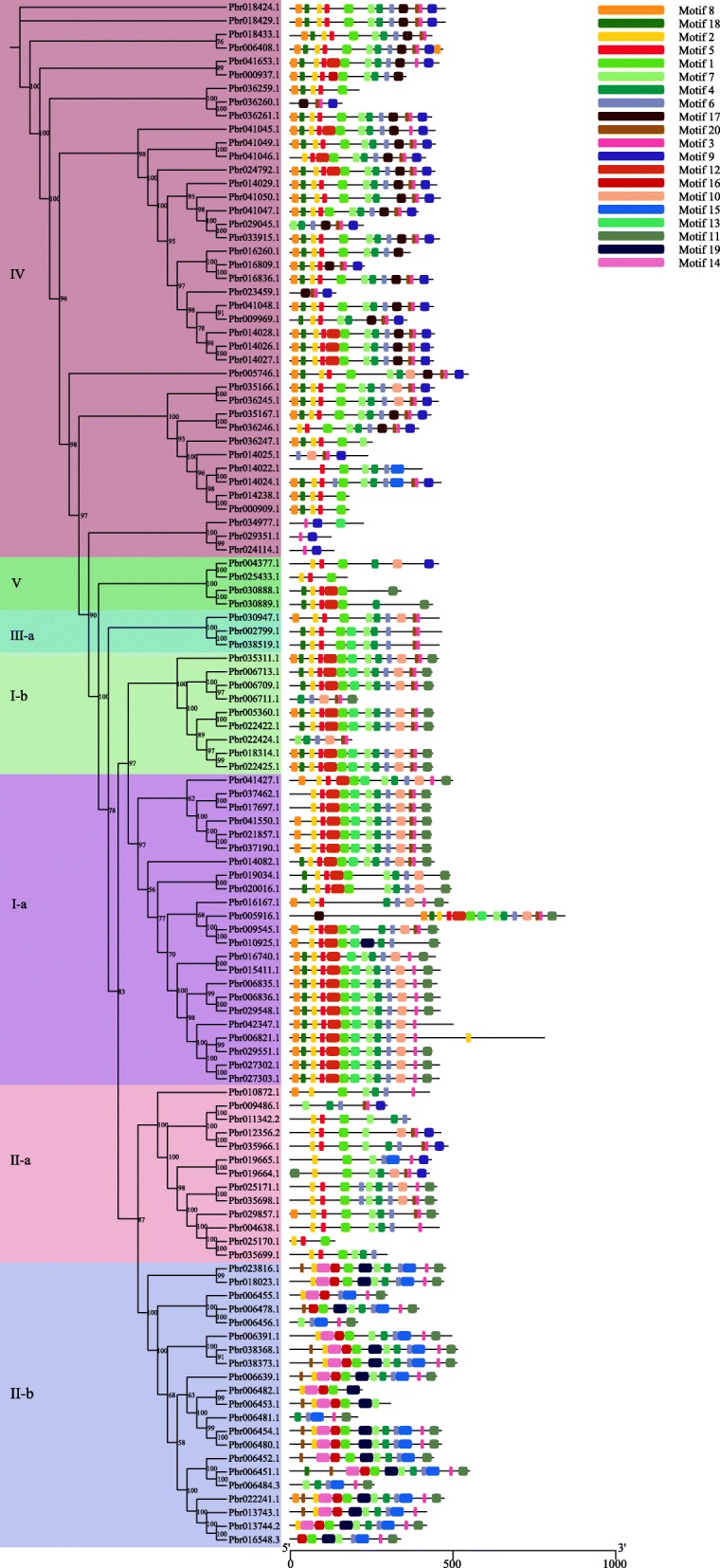
Phylogeny and conserved motifs of *BAHD* genes in pear. MEME tools were used to identify motifs. Different colors represent different motifs

We detected 20 conserved motifs in the BAHD proteins of Chinese white pear using the online software MEME (Fig. 3). All the *BAHD* family members contained motif1 or motif3, and ~ 65.8% (75 of 114) of members contained them both. Based on a gene structural analysis, we determined that motif1 and motif3, with sequences CGGFAIGLSMSHKVADGSSLSTFINSWAE and FYEADFGWGKP, respectively, correspond to domains HXXXD and DFGWG, respectively. Members of subclades I-a, I-b, II-a, II-b, III-a and V do not contain motif17, except for *Pbr005916.1*, and members of the subclades I-a, I-b, II-a, II-b and III-a, as well as clade V, do not contain motif19, except for *Pbr010925.1*. Except for *Pbr005746.1*, *Pbr014025.1*, *Pbr035166.1* and *Pbr036245.1*, members of clade IV do not contain motif10. Motif14 and motif16 were only detected in the Clade II-b. The type and distribution of the conservative motifs of the same subclades were similar, further supporting the evolutionary tree’s classification (Fig. 3). Information on conservative motifs is shown in Additional file 1: Table S2.

### Gene duplication events identified in the pear *BAHD* superfamily and a *BAHD* collinearity analysis of seven Rosaceae species

**Fig. 4:**
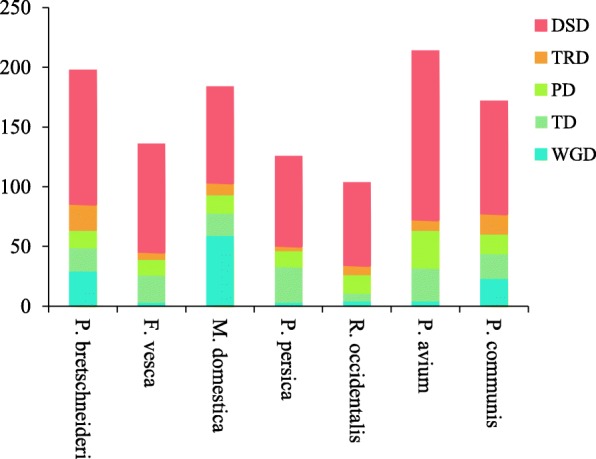
Different modes of gene duplication in *BAHD* families. The *x*-axis represents the species. The *y*-axis represents the number of duplicated gene pairs

Different patterns of gene replication have jointly promoted the evolution of the BAHD family, including whole-genome duplication (WGD) or segmental duplication, tandem duplication (TD), proximal duplication (PD), transposed duplication (TRD) and dispersed duplication (DSD) [34, 35]. We used DupGen_finder software [36] to detect duplicated *BAHD* family gene pairs in seven Rosaceae genomes. All the *BAHD* gene family members were assigned to WGD, PD, TD, TRD or DSD. The number of WGD duplications in Chinese white pear and apple were 29 and 59, respectively, but there were only three in strawberry and peach, four in black raspberry and sweet cherry, and 23 in European pear. The number of DSDs in Chinese white pear, European pear and sweet cherry were 113, 95 and 142, respectively. Additionally, there were 91 in strawberry and 81 in apple, which are more than in peach (76) and black raspberry (70). Genomic rearrangements and gene loss may lead to the large proportion of DSDs in these species. Moreover, the RNA- and DNA-based TRD event can also produce this result [34]. WGDs and DSDs impacted the evolution of the *BAHD* superfamily in Chinese white pear, apple and European pear (Fig. 4). In peach and strawberry TDs and DSDs were the main forces, while PDs and DSDs played major roles in the evolution of black raspberry and sweet cherry. In pear, ~ 57.1% (113 of 198) *BAHD* genes were involved in DSD events, while there were 66.9% (91 of 136) in strawberry, 44.0% (81 of 184) in apple, 60.3% (76 of 126) in peach, 67.3% (70 of 104) in black raspberry, 66.4% (142 of 214) in sweet cherry and 55.2% (95 of 172) in European pear (Additional file 1: Table S3). The results indicated that DSDs were ubiquitous in all the investigated species.

**Fig. 5:**
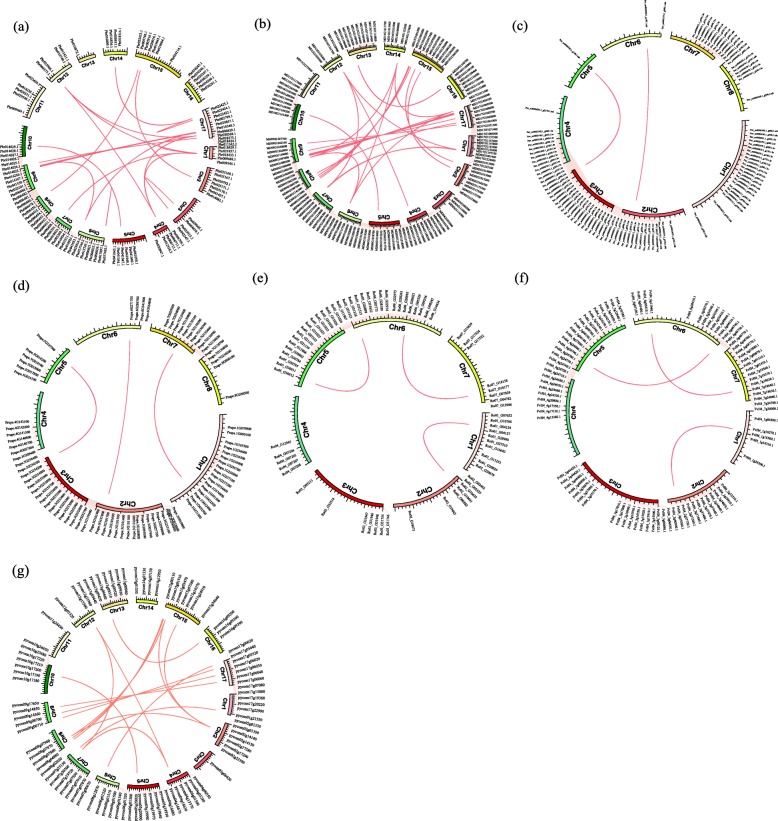
Gene location and collinearity analysis of the *BAHD* gene family. **a**: Chinese white pear; **b**: apple; **c**: sweet cherry; **d**: peach; **e**: black raspberry; **f**: strawberry and **g**: European pear. The genes were located on different chromosomes. Red lines represent the syntenic gene pairs

In addition, we identified intra-genomic synteny blocks for each species [34]. As shown in Fig. 5a, the *BAHD* genes of Chinese white pear are randomly distributed on 17 chromosomes and there is only one gene on chromosome 13. Similarly, the *BAHD* genes were detected as randomly distributed in the other species. We found 78 syntenic gene pairs among the seven Rosaceae species. Of these, 17, 21, and 29 syntenic pairs were identified in European pear (Fig. 5g), Chinese white pear (Fig. 5a) and apple (Fig. 5b), compared with only three in strawberry (Fig. 5f), peach (Fig. 5d) and black raspberry (Fig. 5e) and two in sweet cherry (Fig. 5c) (Additional file 1: Table S4).

### Nonsynonymous (Ka) and synonymous (Ks) substitutions per site, and Ka/Ks analysis for *BAHD* family genes

The stage of evolution for the WGD is usually estimated using Ks [37–39]. In addition to the original WGD [Ks = 1.5–1.8, ~ 140 million years ago (Mya)] (denoted as a γ-paleohexaploidization event) that was shared by core eudicots [40], a more recent WGD was detected in pear and dated to 30–45 Mya (Ks = 0.15 to 0.3) [1]. As shown in Additional file 1: Table S5, Ks values of WGD-derived gene pairs in Chinese white pear ranged from 0.006 to 3.909, and the ranges of Ks values for gene pairs derived from TD, PD, TRD and DSD were 0.001–4.247,0.07–3.670,0.029–4.381 and 0.005–5.066, respectively. Similar results were found in apple. In Chinese white pear, there are nine WGD-derived genes pairs with Ks values that ranged from 0.15 to 0.30, demonstrating that they may be derived from the current WGD (30–45 Mya) [1]. Some other duplicated gene pairs possessed higher Ks values (1.992–3.909), implying that they probably originated from more ancient duplication events. The Ks values of the WGD-derived gene pairs in black raspberry, European pear, peach and sweet cherry were 1.356–2.965, 0.145–4.288, 1.416–4.357 and 1.469–4.210, respectively. The higher Ks values of WGD-derived gene pairs in peach, black raspberry and sweet cherry suggested that they were duplicated and retained from more ancient WGD events, supporting the absence of more recent WGD events in these species.

**Fig. 6:**
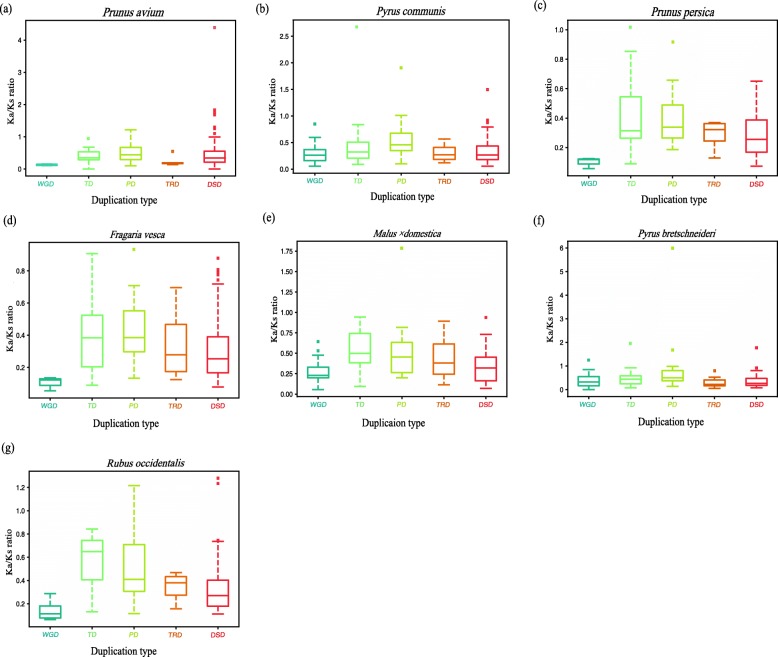
Ka/Ks ratio of seven Rosaceae species. We analyzed the Ka/Ks values using coding sequences. The *x*-axis represents five different duplication categories. The *y*-axis indicates the Ka/Ks ratio. **a**: sweet cherry; **b**: European pear; **c**: peach; **d**: strawberry; **e**: apple; **f**: Chinese white pear and **g**: black raspberry

Deleterious mutations can be removed by negative selection (purifying selection). Conversely, new favorable mutations can be accumulated by positive selection (Darwinian selection) and spread through the population [34]. To detect the selection pressure acting on *BAHD* genes, we analyzed the Ka and Ka/Ks values in the seven Rosaceae species (Additional file 1: Table S5). The direction and magnitude of the selection pressure were inferred based on Ka/Ks ratio (Ka/Ks > 1: positive selection; Ka/Ks = 1: neutral evolution; and Ka/Ks < 1: purifying selection) [41]. The Ka/Ks values of all the *BAHD* gene pairs in strawberry (Fig. 6d), peach (Fig. 6c) and European pear (Fig. 6b) were less than one, indicating that these genes evolved through purifying selection (Fig. 6). Similar results were found in the other four Rosaceae species [the Chinese pear (Fig. 6f), sweet cherry (Fig. 6a), black raspberry (Fig. 6g) and apple (Fig. 6e)], except for a few gene pairs with Ka/Ks values greater than one. The box plots also indicated that the data distributions were concentrated, especially in Chinese white pear, sweet cherry and apple.

### Expression pattern of *BAHD* genes in Chinese white pear

**Fig. 7:**
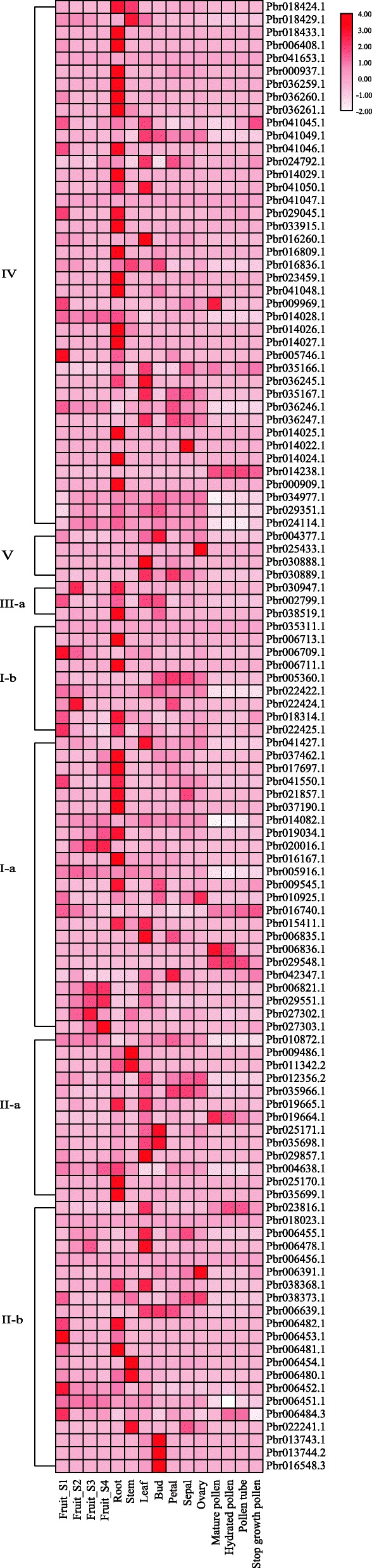
Heat map analysis of *BAHD* genes in pear. RNA-seq data was used to determine the expression patterns of *BAHD* gene. The classes IV–II-b on the left indicate the different clades. Fruit_S1–Fruit_S4 indicate the four distinct stages: on 16th May 2015 (Fruit_S1); 1st July 2015 (Fruit_S2); 31st July 2015 (Fruit_S3); and 29th August 2015 (Fruit_S4). Dark red indicates high expression, and light pink indicates low expression

Based on transcriptome data (Additional file 1: Table S6) from different pear tissues, we determined that most genes in Chinese white pear showed higher expression in roots (Fig. 7), and we discovered that 37 of the *BAHD* genes were expressed in all four stages of fruit development. *Pbr014238.1* was only expressed in the four stages of pollen-tube development, while *Pbr020016.1*, *Pbr027303.1*, *Pbr029551.1*, *Pbr014028.1* and *Pbr006821.1* were highly expressed in the late stage of fruit development (Fruit_S4). Most members of the *BAHD* superfamily showed no expression during the four stages of pollen-tube development.

### Gene expression analyses with qRT-PCR

**Fig. 8:**
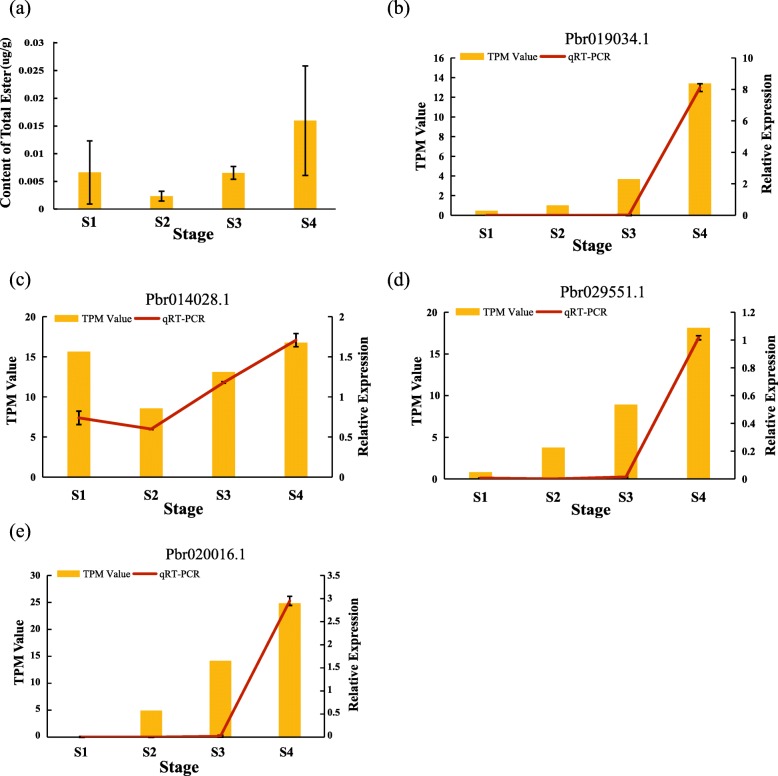
The relative expression levels of several *BAHD* genes among different periods. Tubulin and WD-repeat protein were used as the reference genes to measure expression levels in each period. The *x*-axis indicates the four distinct periods (45DAF, 75DAF, 105DAF and 145DAF). The *y*-axis indicates the relative expression and TPM (Transcripts per million reads) value . Data represent the means ± SDs (*n* = 3). **a**: Content of total ester; **b**: *Pbr019034.1*; **c**: *Pbr014028.1*; **d**: *Pbr029551.1* and **e**: *Pbr020016.1*

Based on the transcriptome expression profiles and the ester content analysis, we selected four potential Chinese white pear genes (*Pbr020016.1*, *Pbr019034.1*, *Pbr014028.1*, and *Pbr029551.1*) that showed strong correlations with total ester content changes during fruit development (Fig. 8a, Additional file 1: Table S7, Additional file 1: Table S8). We used qRT-PCR to examine these candidate genes. The expression patterns of several individual genes were highly correlated with the ester content changes during pear fruit development (Fig. 8). Our results indicated that the expression level of *Pbr014028.1* (Fig. 8c) decreased from S1 (45DAF) to S2 (75DAF), increased sharply from S3 (105DAF) to S4 (145DAF), and then reached a peak value. Surprisingly, three indices of *Pbr014028.1* (Fig. 8c), the relative expression level, the RNA-seq data and the changes in total ester content at all stages exhibited correlated trends. In addition, *Pbr019034.1* (Fig. 8b), *Pbr029551.1* (Fig. 8d) and *Pbr020016.1* (Fig. 8) showed similar expression patterns. They each had a sharp increase from S3 (105DAF) to S4 (145DAF) and reached their peak value in the last period. The results showed that the expression levels of these genes, the RNA-seq data and the changes in total ester contents at all the stages exhibited correlated trends. Therefore, the four genes (*Pbr020016.1*, *Pbr019034.1*, *Pbr029551.1* and *Pbr014028.1*) appear to be important candidate genes for ester synthesis.

**Fig. 9:**
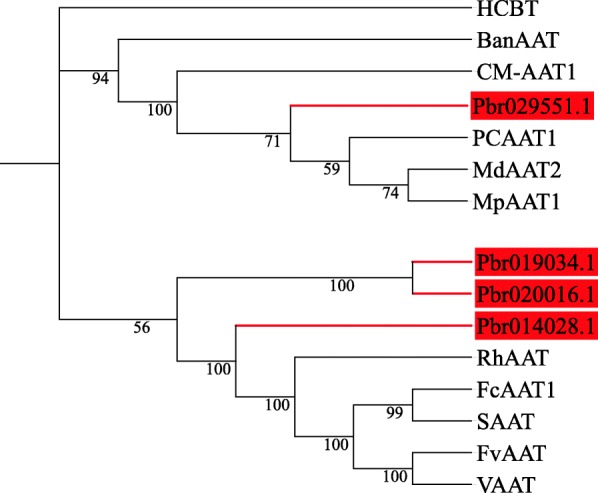
Phylogenetic analysis of candidate genes and 11 function-known *AAT* genes from other species. The maximum-likelihood method was used to construct the phylogenetic tree. The GenBank accession numbers are as follows: *HCBT* (Z84383), *BanAAT* (AX0255O6), *CM-AAT1* (CAA94432), *PCAAT1* (AAS48090), *MdAAT2* (AAS79797), *MpAAT1* (AY707098), *RhAAT* (AAW31498), *FcAAT1* (FJ548610), *SAAT* (AAG13130), *FvAAT* (AAN07090), *VAAT* (AX025504)

To further investigate this result, four *BAHD* genes (*Pbr020016.1*, *Pbr019034.1*, *Pbr014028.1* and *Pbr029551.1*) from Chinese white pear and 11 biochemically characterized *AAT* genes from other species were used to construct a maximum-likelihood tree. As seen in Fig. 9, we found that these pear *BAHD* genes shared a high homology with the reported *AAT* genes. Thus, we speculated that these genes might have a strong correlation with ester synthesis.

## Discussion

The *BAHD* superfamily has members in various ester synthetic pathways. Here, by detecting their HXXXD and DFGWG motifs using a HMMER search, we identified 717 *BAHD* genes from seven Rosaceae species. Similar to the results of the classification of *BAHD* genes in *Arabidopsis* [28], *BAHD* family protein-encoding genes of pear were classified into five clades (I, II, III-a, IV and V)*.* In addition, a conserved motif analysis of these *BAHD* amino acid sequences showed that the type and distribution of the conservative motifs among the same subfamily were similar, indicating that the classification results are authentic. All the *BAHD* family members contained motif1 (HXXXD) or motif3 (DFGWG), and ~ 65.8% (75 of 114) of members contained them both. Thus, we hypothesized that the motifs (1 and 3) may be related to catalytic activity. The HXXXD catalytic motif is located in the solvent channel, and it can also deprotonize oxygen or nitrogen atoms on specific receptor substrates [13, 17]. The DFGWG motif is essential to the reaction but located far from the active site [13, 17, 42, 43]. Combining the phylogenetic tree classification with the results of previous studies, we speculated that the *BAHD* genes that are closely related to the pear volatile ester content, such as *AAT*, belonged to clades I and IV, and the *AAT* are involved in the synthesis of esters, which are involved in determining the aromas of flowers and fruits [44]. At present, the functions of many *AAT* genes in fruit trees have been identified, such as *PpAAT1* in peach [45], *FaAAT2* and *SAAT* in cultivated strawberry [25, 26], *MpAAT1* in apple [25] and *CmAAT1*,3,4 in melon [46, 47].

Gene duplication patterns can be generally divided into five types, WGD, PD, TD, DSD and TRD [34, 35]. Each pattern of gene duplication contributes differentially to gene family expansion [48]. It is estimated that 90% of the gene increase in the *Arabidopsis thaliana* pedigree is due to the result of WGDs [49]. WGD, TD and DSD are the main features of eukaryotic genome evolution and mainly drive the development of new functions in the genome and genetic evolutionary systems [50, 51]. Gene families, such as *SWEET* and Hydroxycinnamoyl transferases, expanded primarily through WGD and DSD [52, 53]. TD was the major force in the expansion of the *AP2/ERF* and *WRKY* gene families [54, 55]. Two gene families, Aluminum-activated malate transporters and heat-shock transcription factors were amplified by WGD and DSD [34, 39]. The numbers of *BAHD* genes identified in seven plants from the Rosaceae family are different because of recent WGD events and ancient polyploid events. In this study, DSD and WGD were determined to be the main forces that expanded the *BAHD* genes in apple, European pear and Chinese white pear. The numbers of *BAHD* genes that had undergone WGD and DSD were the largest, while the numbers of genes that underwent other replication modes were relatively small. In addition, because peach, sweet cherry, strawberry and sweet cherry have not undergone recent WGD events, the number of DSDs account for a large proportion of the gene duplications. Accordingly, WGD patterns account for large proportions in apples and pears, perhaps because they have experienced two rounds of WGD [40]. In addition, based on the Ka, Ks and Ka/Ks analysis, we discovered that in peach, black raspberry and sweet cherry the Ks values of WGD-derived gene pairs were greater than 1.3, suggesting that they were duplicated and retained from more ancient WGD events. This further supported the absence of more recent WGD events in these species. We also found that all the *BAHD* gene pairs in strawberry, peach and European pear had Ka/Ks < 1, indicating that they have experienced strong purifying selection.

The diversity of the biochemical functions of the *BAHD* genes has been determined in many species. For example, the *BAHD* acyltransferase gene can catalyze the last step in cocaine biosynthesis [56], and the protein plays an important role in plant innate immunity [57]. Furthermore, *AAT* gene functions have been determined in several species. For instance, the *AAT* gene in apple, *MdAAT2*, may provide resistance to stress and can change the volatile compound profile [58]. In Mountain papaya (*Vasconcellea pubescens*), *VpAAT1* is involved in ester biosynthesis [59]. Despite the diverse functions of the *BAHD* gene family members, we focused on their roles in ester synthesis. Volatile esters produced by these *AAT* genes usually promote the recognition of plants as food because they contribute to the “fruity” aroma of edible fruits, and some esters are also the key flavor or smell component of a particular plant [60]. In this study, our RNA-seq data revealed that 37 *BAHD* genes were detected at various expression levels in the four stages of pear fruit development. Surprisingly, though lacking motif3 (DFGWG), the *Pbr019034.1* and *Pbr020016.1* genes were both still expressed at all the stages. Based on transcriptome expression profiles combined with the ester content analysis, four potential genes (*Pbr020016.1*, *Pbr019034.1*, *Pbr014028.1* and *Pbr029551.1*) belonging to clades I and IV and that showed strong correlations with ester content changes during fruit ripening, were selected for further qRT-PCR analysis. The assessed expression patterns of the four genes, *Pbr020016.1*, *Pbr029551.1*, *Pbr019034.1*, and *Pbr014028.1*, were basically consistent with the RNA-seq data.

## Conclusions

Here, we identified 717 *BAHD* genes from seven species, and among them, 114 belonged to Chinese white pear. We divided the *BAHD* superfamily into five large clades according to the classification results from model plants. *BAHD* genes expanded in seven Rosaceae species and experienced strong purifying selection except for a few gene pairs with Ka/Ks values greater than one. Finally, after RNA-seq data and qRT-PCR analysis, *Pbr020016.1*, *Pbr029551.1*, *Pbr019034.1* and *Pbr014028.1* were found to be candidate genes related to ester synthesis*.* These results provide a foundation for further studies of the molecular mechanisms underlying aroma biosynthesis and release.

## Methods

### Source of plants

The ‘Dangshan suli’ [1] fruit were picked at four different developmental stages from the pear germplasm orchard of the Center of Pear Engineering Technology Research located at Jiangpu in Nanjing. The fruit samples were collected on 26th May (45 DAF), 27th June (75 DAF), 28th July (105 DAF) and 6th Sep (145 DAF) in 2017. Previously published RNA-seq data was used to analyze the expression patterns of ‘Dangshan suli’ *BAHD* [61, 62]. All the samples were ground in liquid nitrogen and stored at − 80 °C. The genome annotation files and genome sequences of pear were collected from the Nanjing Agricultural University pear genome project website (http://peargenome.njau.edu.cn), and the other four Rosaceae species sequences (*Fragaria vesca, Pyrus communis, Prunus avium* and *Rubus occidentalis*) were downloaded from the Genome Database for Rosaceae (http://www.rosaceae.org). The sequences of apple (*M. domestica*) and peach (*P. persica*) were downloaded from Joint Genome Institude (http://www.jgi.doe.gov/).

### Sequence identification and collection

To identify putative *BAHD* genes from pear and the six other species, peach, apple, strawberry, European pear, sweet cherry and black raspberry, several approaches were employed. Using a *BAHD* family characteristic domain (Pfam: PF02458) as a query sequence in accordance with the HMM configuration file (PF02458) of *BAHD*, we searched for candidate genes using the HMM with E-values <1e^− 10^. We downloaded the HMM profile (PF02458) from the Pfam website (https://pfam.xfam.org/). The online site SMART (http://smart.embl-heidelberg.de/) was used to determine the domain PF02458 of *BAHD*, and then their HXXXD and DFGWG motifs were inspected. The online website MAFFT (https://mafft.cbrc.jp/alignment/server/) was used for multiple sequence alignments, and the comparison results were download to GeneDoc (http://www.pscedu/biomed/genedoc).

### Phylogenic analysis

We use the online website MAFFT for multiple sequence alignments, and then upload the comparison results to IQ-TREE (http://www.cibiv.at/software/iqtree) [63]. The ML phylogenetic trees were constructed with bootstraps of 1000 using IQ-TREE. To verify the ML phylogenetic tree, a NJ phylogenetic tree was constructed using MEGA 7.0, with a bootstrap of 1000 [64].

### Conservative motif analysis of *BAHD* family members

The conservative motifs were analyzed using the online software MEME (http://meme.sdsc.edu/meme4_3_0/intro.html). The maximum value of the motif was set to 20, and the motif length was set between six and 200.

### Identification of gene duplication modes and a collinearity analysis

A gene duplication analysis among seven Rosaceae genomes was performed, and the method developed by PGDD (http://chibba.agtec.uga.edu/duplication/) was carried out locally [65]. First, the chromosomal locational information of the *BAHD* gene family members and related gene pair information were obtained from the genome annotation files of each species, and then the localization and synteny of the *BAHD* genes were determined using TB-tools software [66]. MCScanX was further used to identify different duplication patterns in the *BAHD* superfamily [67].

### Calculating Ka and Ks

Ka, Ks and the Ka/Ks ratio were calculated using the calculate_Ka_Ks_pipeline (https://github.com/qiaoxin/Scripts_for_GB/tree/master/calculate_Ka_Ks_pipeline) [36]. In brief, the coding sequences and gene pairs were prepared first. Then, we ran the computing_Ka_Ks_pipe.pl script to automatically perform multiple alignments using MAFFT software and to convert the AXT format, which was used as input for KaKs_Calculator [68], to calculate Ka and Ks using the GMYN model. Finally, the readable results, including Ka, Ks, Ka/Ks and *P*-value, were generated.

### Expression analysis of *BAHD* in 15 samples of *P. bretschneideri* and a qRT-PCR analysis

The RNA-seq data of 15 ‘Dangshansuli’ samples were used to analyze the expression patterns of ‘Dangshansuli’ *BAHD,* including Fruit_S1, Fruit_S2, Fruit_S3, Fruit_S4 (obtained from the NCBI biproject PRJNA563942), Root (data not shown), Stem, Leaf, Bud, Petal, Sepal, Ovary, Mature pollen, Hydrated pollen, Pollen tube and Stop growth pollen [61, 62]. Then, the gene expression heatmap was drawn using TB-Tools [66].

Based on the correlation analysis between transcriptome and ester content data of pear fruit during four developmental stages, we selected five possible *AAT* genes for qRT-PCR. The primers of all the possible genes were designed using Primer Premier 6.0. Total RNA was extracted using a Plant Total RNA Isolation Kit Plus (Fuji, China), and cDNA was synthesized using TransScript One-Step gDNA Removal and cDNA Synthesis SuperMix (TransGen, China). The PCR mixture was as follows: 1 μl of sense and anti-sense primer (10 μM), 5 μl fluorescent dye, 1 μl template and 3 μl sterilized water. qRT-PCR was performed using a LightCycler 480 SYBRGREEN I Master (Roche, USA). We ran the PCR reaction under the following conditions: 10 min at 95 °C, followed by 45 cycles of 95 °C for 3 s, 60 °C for 10 s and 72 °C for 30 s. Then, the 2^−△△ct^ method was used to determine the relative expression with the reference genes being tubulin and WD-repeat protein (Additional file 1: Table S9).

### Analysis of volatile aroma compounds

The SPME-GC method was used to determine volatile aromas from Chinese white pear. The concentrations of components (μg/g) = [peak area of component/peak area of internal standard × the concentration of internal standard (g/ml) × 5 μl]/mass of sample (g). The concentration of the internal standard 3-nonanone was 0.82 × 10^− 3^ g/ml [5]. The SPME fibers used in this study to adsorb volatile organic compounds were coated with a 65 μm thickness of polydimethylsiloxane–divinylbenzene (65 μm PDMS/DVB; Supelco Co, Bellefonte, PA, USA) [6]. The fibers were activated before sampling according to the manufacturer’s instructions. In each extraction, 5 g of pulp were placed into a 20-ml screw-cap headspace brown vial and then add 5 ml of NaCl solution (0.36 g/ml), finally, add 5 ul 3-nonanone solution (0.82 g/l) as internal standard [6]. The mixture was placed in a constant-temperature water bath at 40 °C while the SPME fibers were exposed to the headspace of the sample for 30 min to adsorb the analytes, and then introduced into the heated inlet of the chromatograph for desorption at 250 °C for 5 min in splitless mode. The specific GC-MS conditions are as follows: The organic extract was analysed with a Bruker 320 mass selective detector coupled to a Bruker 450 gas chromatograph, equipped with a 30 m × 0.25 um × 0.25 mm BR-5 MS (5% phenyl-polymethylsiloxane) capillary column. Helium was used as the carrier gas at a flow of 1.0 ml/min. The injector and detector temperature were 250 and 280 °C, respectively. Mass spectra were recorded at 70 eV in electron impact (EI) ionization mode. The temperatures of the quadrupole mass detector and ion source were 150 and 230 °C, respectively. The temperature of the transfer line was 280 °C. Finally, volatile organic compounds were initially identified by comparing the mass spectra of the samples with the data system library (NIST 2013) [6].

## Supplementary information

**Additional file 1: Table S1.** Gene lists and basic feature of all identified *BAHD* genes. **Table S2.** Motif sequences identified by MEME tools in pear *BAHD*s. **Table S3.** Information of gene duplication events identified in all identified *BAHD* genes. **Table S4.** Collinearity relationship among *BAHD* genes in the same species. **Table S5.** Ka, Ks and Ka/Ks value of duplicated gene pairs among *BAHD*. **Table S6.** The TPM value of *BAHD* genes in pear. **Table S7.** Content of total ester at different developmental stages of Chinese white pear. **Table S8.** The list of molecules identified as esters. **Table S9.** Primers for qRT-PCR of candidate genes in Chinese white pear *BAHD* gene family.

**Additional file 2: Figure S1.** Phylogenetic analysis of *BAHD* from *Arabidopsis, Pyrus bretschneideri* and *Populus.* The software MEGA 7.0 was used to construct the phylogenetic tree. The amino-acid sequences of *Arabidopsis* and *Populus* were obtained from phytozome (https://phytozome.jgi.doe.gov/pz/portal.html#).

**Additional file 3: Figure S2.** Phylogenetic analysis of *BAHD* from *Arabidopsis, Pyrus communis* and *Populus.* The software MEGA 7.0 was used to construct the phylogenetic tree. The amino-acid sequences of *Arabidopsis* and *Populus* were obtained from phytozome (https://phytozome.jgi.doe.gov/pz/portal.html#).

## Data Availability

The pear genome datasets used during the current study are available in our pear center website (http://peargenome.njau.edu.cn/), the other four Rosaceae species sequences (*Fragaria vesca*, *Pyrus communis*, *Prunus avium* and *Rubus occidentalis*) were downloaded from the GDR (http://www.rosaceae.org). The sequence of apple (*Malus domestica*) and peach (*Prunus persica*) were collected from JGI (http://www.jgi.doe.gov/). The RNA-seq data were obtained from the NCBI database (https://www.ncbi.nlm.nih.gov/).

## Abbreviations

AAT: Alcohol acyltransferase
DSD: Dispersed duplication
HMM: Hidden Markov Model
ML: maximum-likelihood
Mya: Million years ago
NJ: neighbor-joining
PD: Proximal duplication
TD: Tandem duplication
TRD: Transposed duplication
WGD: Whole-genome duplication
