# Supplementary figures and images for: Genome-wide comparative analysis of the BAHD superfamily in seven Rosaceae species and expression analysis in pear (Pyrus bretschneideri)
Source: BMC Plant Biol. 2020 Jan 8;20:14. doi: 10.1186/s12870-019-2230-z (PMC6950883; doi:10.1186/s12870-019-2230-z)

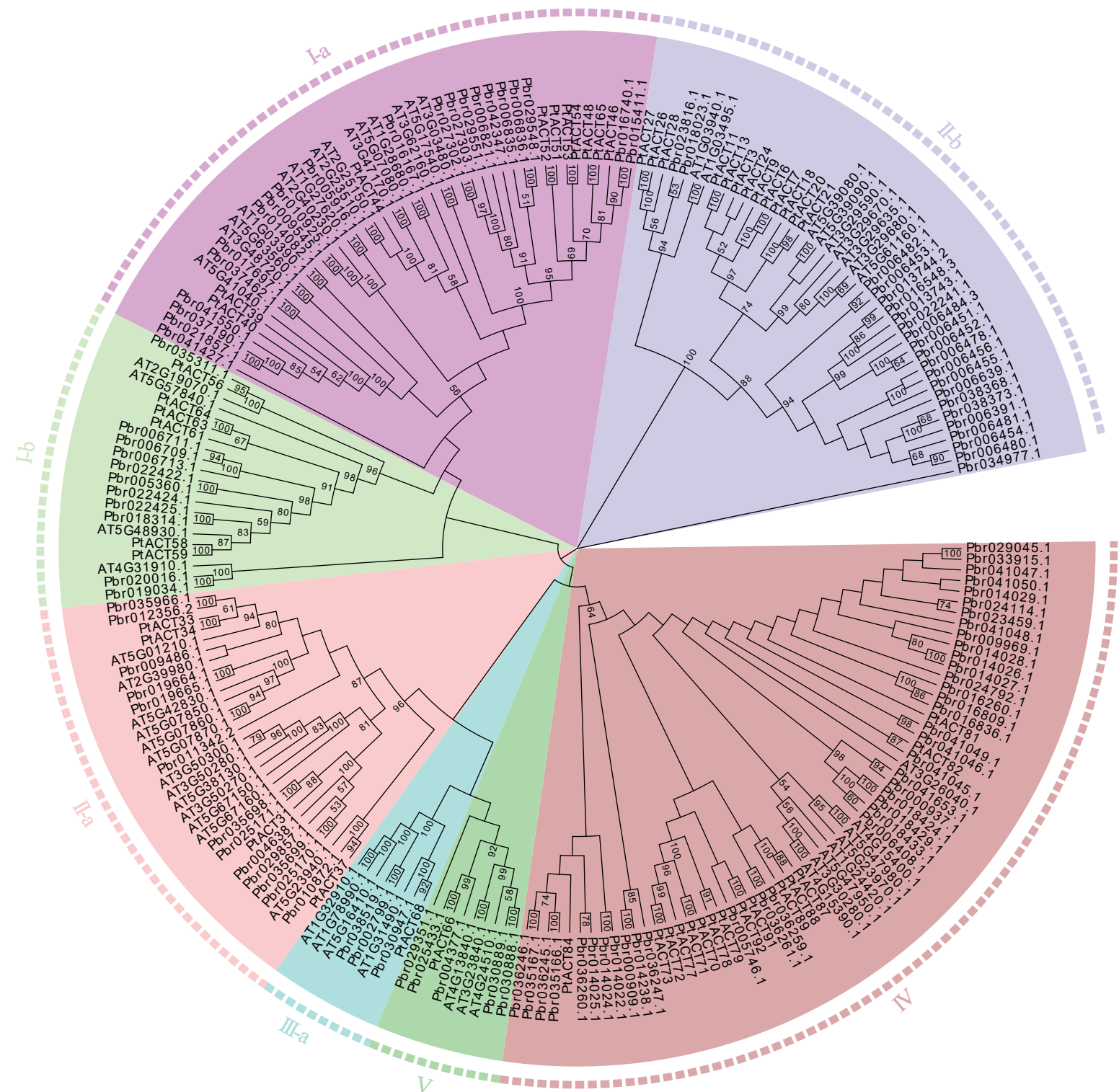

Supplement: Supplementary file 2 — Additional file 2: Figure S1. Phylogenetic analysis of BAHD from Arabidopsis, Pyrus bretschneideri and Populus. The software MEGA 7.0 was used to construct the phylogenetic tree. The amino-acid sequences of Arabidopsis and Populus were obtained from phytozome (https://phytozome.jgi.doe.gov/pz/portal.html#). [file 12870_2019_2230_MOESM2_ESM.pdf]

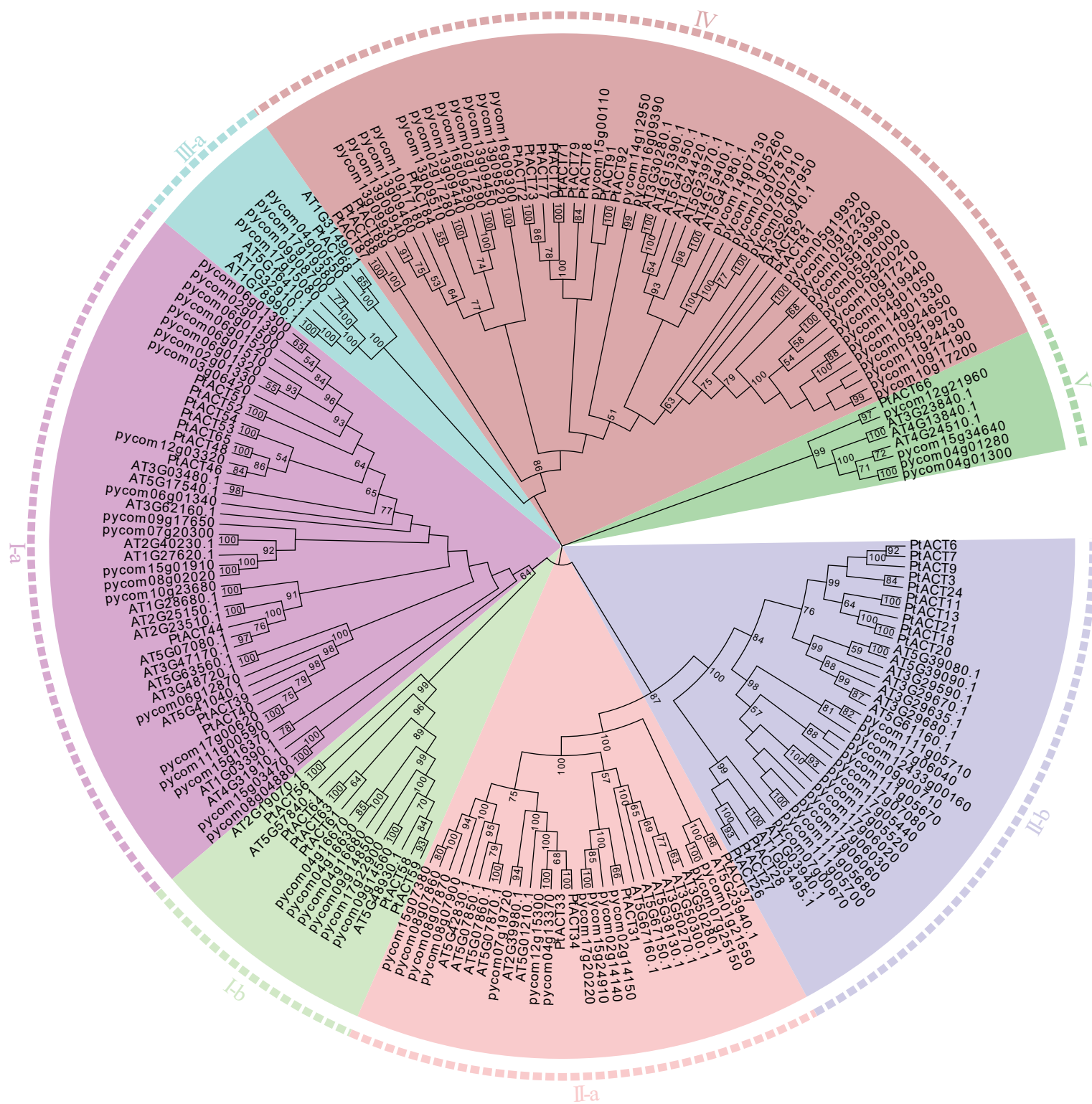

Supplement: Supplementary file 3 — Additional file 3: Figure S2. Phylogenetic analysis of BAHD from Arabidopsis, Pyrus communis and Populus. The software MEGA 7.0 was used to construct the phylogenetic tree. The amino-acid sequences of Arabidopsis and Populus were obtained from phytozome (https://phytozome.jgi.doe.gov/pz/portal.html#). [file 12870_2019_2230_MOESM3_ESM.pdf]
